# Structural remodelling of the sinoatrial node in obese old rats

**DOI:** 10.1016/j.yjmcc.2009.08.023

**Published:** 2010-04

**Authors:** J. Yanni, J.O. Tellez, P.V. Sutyagin, M.R. Boyett, H. Dobrzynski

**Affiliations:** a Cardiovascular Medicine, School of Medicine, University of Manchester, Core Technology Facility, 46 Grafton Street, Manchester M13 9NT, UK; b Department of Morphology, Russian State Medical University, Moscow, Russia

**Keywords:** Sinus node, Ageing, Sick sinus syndrome, Ion channels, Extracellular matrix

## Abstract

During ageing, the function of sinoatrial node (SAN), the pacemaker of the heart, declines, and the incidence of sick sinus syndrome increases markedly. The aim of the study was to investigate structural and functional remodelling of the SAN during ageing. Rats, 3 and 24 months old (equivalent to young adult and ∼ 69-year-old humans), were studied. Extracellular potential recording from right atrial preparations showed that (as expected) the intrinsic heart rate was slower in the old animals. It also showed a shift of the leading pacemaker site towards the inferior vena cava in the old animals. Consistent with this, intracellular potential recording showed that slow pacemaker action potentials were more widespread and extended further towards the inferior vena cava in old animals. Immunohistochemistry demonstrated that SAN tissue expressing HCN4, but lacking the expression of Na_v_1.5 (lack of Na_v_1.5 explains why pacemaker action potential is slow), was also more widespread and extended further towards the inferior vena cava in the old animals. Immunolabelling of caveolin3 (expressed in cell membrane of cardiac myocytes) demonstrated that there was a hypertrophy of the SAN cells in the old animals. Histology, quantitative PCR, and immunohistochemistry revealed evidence of a substantial age-dependent remodelling of the extracellular matrix (e.g. ∼ 79% downregulation of genes responsible for collagens 1 and 3 and ∼ 52% downregulation of gene responsible for elastin). It is concluded that the age- (and/or obesity-) dependent decline in SAN function is associated with a structural remodelling of the SAN: an enlargement of the SAN, a hypertrophy of the SAN cells, and a remodelling of the extracellular matrix.

## 1 Introduction

Cardiovascular disease is a leading cause of morbidity and mortality and it occurs with increasing incidence in the elderly [1]. The function of the sinoatrial node (SAN), the pacemaker of the heart, declines with age – there is a decrease in the intrinsic heart rate (heart rate in absence of autonomic tone) and an increase in the SAN conduction time [2]. There is also a strong correlation between ageing and the incidence of sick sinus syndrome [2]. Manifestations of sick sinus syndrome include bradycardia, SAN arrest, and SAN exit block [2]. The mechanisms underlying the age-dependent decline in SAN function are unclear.

There is evidence that ageing is associated with a structural remodelling of the SAN. First, there is electrophysiological evidence of an enlargement of the SAN: Alings and Bouman [3] showed that, in the rabbit and cat, the SAN region with a ‘slow action potential’ increases with age—it has been suggested that this is responsible for the decline in SAN function [4]. There is a slow action potential in the centre of the SAN, because there is little or no Na^+^ current, *I*_Na_, and the action potential has a slow upstroke generated by the L-type Ca^2+^ current, whereas in working myocardium the action potential has a fast upstroke generated by *I*_Na_. Secondly, there is evidence of a change in the interstitium (fibroblasts and extracellular matrix). Throughout the heart, ageing is associated with changes in the interstitium, which functions as a support for cardiac myocytes and blood vessels and also maintains myocardial mass, structure, and function [5]. Histologically, the SAN is distinguished from the surrounding atrial muscle (and working myocardium in general) by a remarkably large amount of interstitium (for example, up to 75%–90% of SAN volume in cat [6]). The function of the interstitium in the SAN is still unclear; it may serve as a mechanical support and give the SAN the stiffness that it lacks because of the poor development of the contractile apparatus in SAN cells [7]. It has been suggested that the age-dependent decline in SAN function is the result of a loss of nodal cells and a compensatory proliferation of the interstitium [8,9]. The aim of this study was to study the changes in the structure and function of the SAN during ageing.

## 2 Materials and methods

Right atrial preparations from male Wistar-Hanover rats aged 3 and 24 months (equivalent to young adult and ∼ 69-year-old humans, respectively [10]) were used. SAN function was studied using extracellular potential and intracellular action potential recording. Protein and mRNA expression was studied using immunohistochemistry and quantitative PCR (qPCR), respectively. Collagen protein was studied using Picro Sirius red staining. The methods are described in full in the Data Supplement.

## 3 Results

Old animals were heavier than young animals and their hearts were also heavier (Data Supplement, Fig. S1). However, there was a significant decrease in the heart-to-body weight ratio (Data Supplement, Fig. S1). In the anaesthetised rat, the normal heart rate is not significantly different between young and old animals (aged 3 and 25 months) [10]. The normal heart rate is set by the autonomic nervous system, and therefore, it is important to measure the intrinsic heart rate. This was measured in isolated right atrial preparations (using extracellular potential recording from dorsal epicardial surface). The intrinsic heart rate in old animals was slower (i.e. spontaneous cycle length was longer) than in young animals (Data Supplement, Fig. S1). The spontaneous cycle length was 297 ± 9 ms (*n* = 7) in young animals and 351 ± 13 ms (*n* = 6) in old animals (*t* test; P < 0.05).

### 3.1 Age-dependent increase in sensitivity to blockade of Na_v_1.5 and HCN4

**Fig. 1:**
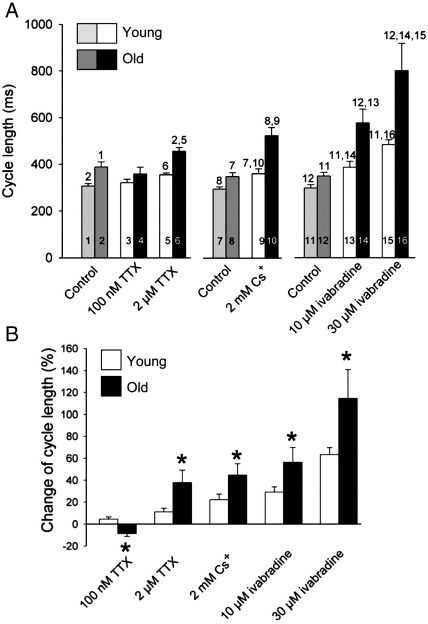
Effect of ion channel blockers on spontaneous cycle length of right atrial preparations from young and old animals. (A) Cycle length: under control conditions and after application of 100 nM TTX (blocker of Na_v_1.1) and subsequent application of 2 μM TTX (blocker of Na_v_1.5) (1–6); under control conditions and after application of 2 mM Cs^+^(blocker of HCN4) (7–10); and under control conditions and after application of 10 and 30 μM ivabradine (another blocker of HCN4) (11–16). The numbers indicate significant differences from the appropriately numbered bars (P < 0.05; one-way ANOVA). (B) Mean (+ SEM; *n* = 6) percentage change in cycle length after blockade of Na_v_1.1 by 100 nM TTX, blockade of Na_v_1.5 by subsequent application of 2 μM TTX, and blockade of HCN4 by 2 mM Cs^+^ and 10 and 30 μM ivabradine in young and old animals. ⁎Significantly different (P < 0.05) from corresponding data from young animals (one-way ANOVA).

The age-dependent decline in SAN function has been suggested to be the result of a decline in *I*_Na_ (see Introduction). This is a possibility, because blockade of *I*_Na_ can slow pacemaking and familial sick sinus syndrome has been linked to mutations in Na_v_1.5 (main ion channel responsible for *I*_Na_), although paradoxically there is little or no *I*_Na_ and Na_v_1.5 in the centre of the SAN [2]. Another possibility is that it is the result of a decline in the funny current, *I*_f_, because blockade of *I*_f_ slows pacemaking and familial sick sinus syndrome has also been linked to mutations in HCN4 (main ion channel responsible for *I*_f_) [2]; *I*_f_ and HCN4 are present in the SAN [2]. To test these possibilities, the effect of blockade of Na_v_1.5 and HCN4 on the spontaneous activity of right atrial preparations was investigated—if the age-dependent decrease in the intrinsic heart rate is the result of a decline in either *I*_Na_ or *I*_f_, blockade of the corresponding ion channel should have a smaller effect on the heart rate in the older animal. TTX was used to block Na_v_1.5. ‘Neuronal Na^+^ channels’, such as Na_v_1.1, which have a high sensitivity to TTX, as well as the cardiac Na^+^ channel, Na_v_1.5, which has a low sensitivity to TTX, are responsible for *I*_Na_ [11]. Blockade of neuronal Na^+^ channels by 100 nM TTX caused small changes in cycle length (significantly different in young and old preparations; Fig. 1). The subsequent application of 2 μM TTX to block Na_v_1.5 caused a slowing of pacemaking in both young and old preparations (Fig. 1A) – however, the percentage increase of the spontaneous cycle length was significantly greater in the old preparations (Fig. 1B). HCN4 was blocked by the application of 2 mM Cs^+^ or 10 μM ivabradine. Blockade of *I*_f_ by 2 mM Cs^+^ is nearly selective and complete [12]. In rabbit SAN cells at least, 10 μM ivabradine causes 81% blockade of *I*_f_ and a slight decrease in *I*_Ca,L_ and *I*_K,r_ [13]. Both 2 mM Cs^+^ and 10 μM ivabradine caused a slowing of pacemaking in both young and old preparations (Fig. 1A) – however, the percentage increase of the spontaneous cycle length was significantly greater in the old preparations (Fig. 1B). Thirty micromolars of ivabradine was also used – the slowing of pacemaking was greater in both young and old preparations, but the effect was still significantly greater in the old preparations (Fig. 1). Thirty micromolars of ivabradine is expected to cause greater blockade of *I*_f_ (93%); however, the non-specific effects of 30 μM ivabradine will be greater [13] (in patients, target plasma concentration of ivabradine is only ∼ 0.7 μM). In summary, there is an age-dependent increase rather than decrease in the sensitivity of the pacemaker activity of the SAN to blockade of Na_v_1.5 and HCN4, and therefore, it is unlikely that the age-dependent decrease in the intrinsic heart rate is the result of a decline in *I*_Na_ or *I*_f_.

### 3.2 Age-dependent increase in pacemaker tissue with ‘slow action potential’

Two lines of evidence showed that there was an age-dependent increase in pacemaker tissue. In both young and old animals, the leading pacemaker site (determined by extracellular potential recording) was located next to the atrial muscle of the crista terminalis. In all young animals, the leading pacemaker site was located near the superior vena cava, whereas in old animals, the position of the leading pacemaker site was generally more inferior and located closer to the inferior vena cava: in young animals, the leading pacemaker site was 1.0 ± 0.4 mm from the superior vena cava, whereas in old animals, it was 2.5 ± 0.4 mm (24 ± 11% and 57 ± 12%, respectively, of the distance between superior and inferior vena cava) (Table 1).

**Fig. 2:**
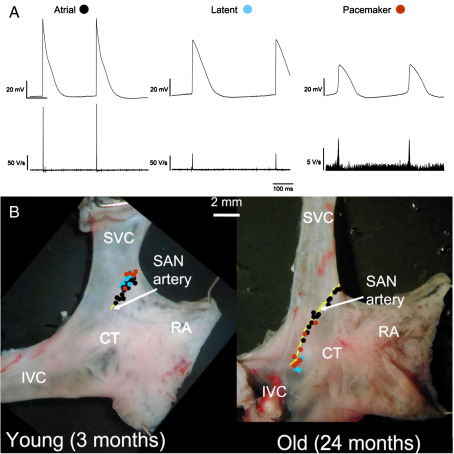
Pacemaker tissue is more widespread in old animals. (A) Typical intracellular action potential recordings (top) and rate of change of membrane potential (bottom) from atrial, latent pacemaker, and true pacemaker cells. Recordings shown were obtained from different animals (one young and two old animals); therefore, no inferences can be drawn concerning activation times. (B) Photographs of dorsal surface of right atrial preparations from young and old animals. Black circles, sites at which atrial action potentials were recorded. Blue circles, sites at which latent pacemaker action potentials were recorded. Red circles, sites at which true pacemaker action potentials were recorded. CT, crista terminalis; IVC, inferior vena cava; RA, right atrial free wall; SVC, superior vena cava. Yellow, SAN artery.

Intracellular action potential recordings from the dorsal epicardial surface of right atrial preparations revealed three types of cell (Fig. 2A): first, atrial cells with a ‘fast action potential’. Atrial cells had a stable resting potential and an action potential with a fast upstroke, large amplitude, and short duration; in young preparations, the maximum upstroke velocity (d*V*/d*t*_max_) was 131 ± 8 V/s, the action potential amplitude was 88 ± 1 mV, and the action potential duration at 15% repolarization (APD_15_) was 6 ± 0.5 ms (*n* = 6; Fig. 2A). Secondly, ‘true’ pacemaker cells with a ‘slow action potential’. The cells had a prominent pacemaker potential, a smooth transition from the pacemaker potential to the action potential, and an action potential with a slow upstroke, small amplitude, and long duration; in young preparations, d*V*/d*t*_max_ was 11 ± 1 V/s, action potential amplitude was 45 ± 2 mV, and APD_15_ was 29 ± 2 ms (*n* = 6; Fig. 2A). Thirdly, latent pacemaker cells with a modest pacemaker potential, an abrupt transition from the pacemaker potential to the action potential, and an action potential with an intermediate upstroke velocity, amplitude, and duration; in young preparations, d*V*/d*t*_max_ was 42 ± 4 V/s, action potential amplitude was 60 ± 2 mV, and APD_15_ was 20 ± 1 ms (*n* = 6; Fig. 2A). Fig. 2B shows the distribution of the three cell types in preparations from a young and old animal. As in a previous study [14], the pacemaker cells were distributed along the SAN artery next to the crista terminalis. In young animals, true and latent pacemaker cells were generally located in a small region near the superior vena cava, whereas in old animals, they were more widely distributed and occurred more inferiorly (Fig. 2B), consistent with the location of the leading pacemaker site as discussed above. In young animals, pacemaker tissue extended along the SAN artery for 1.7 ± 0.2 mm, whereas in old animals, it extended for 3.1 ± 0.3 mm (47% and 65%, respectively, of distance between superior and inferior vena cava) (Table 1).

During ageing, as discussed elsewhere [10], there was no significant change in d*V*/d*t*_max_ and APD_15_, but there was a significant increase (22%) in action potential duration at 75% repolarization in true and latent pacemaker cells.

### 3.3 Age-dependent increase in pacemaker tissue lacking expression of Na_v_1.5 and expressing HCN4

**Fig. 3:**
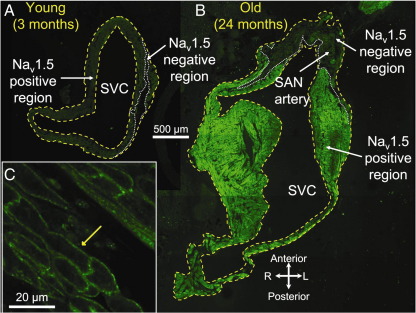
Na_v_1.5 expression. (A and B) immunolabelling of Na_v_1.5 (green signal) in sections through head of SAN in young (A) and old (B) animals. Yellow dashed lines, border of tissue sections; white dashed lines, border between Na_v_1.5 expressing and non-expressing regions. (C) High-power image of immunolabelling of Na_v_1.5 in atrial muscle. Yellow arrow highlights immunolabelling of Na_v_1.5 in cell membrane. L, left; R, right; SVC, superior vena cava.

**Fig. 4:**
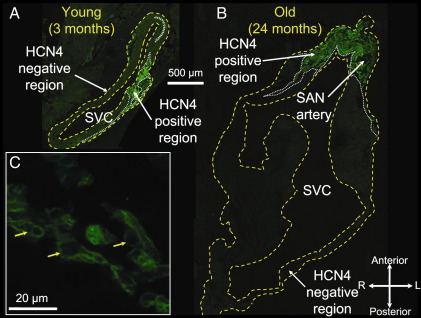
HCN4 expression. (A and B) immunolabelling of HCN4 (green signal) in sections through head of SAN in young (A) and old (B) animals. Yellow dashed lines, border of tissue sections; white dashed lines, border between HCN4 expressing and non-expressing regions. (C) High-power image of immunolabelling of HCN4 in SAN. Yellow arrows highlight immunolabelling of HCN4 in cell membrane. L, left; R, right; SVC, superior vena cava.

The SAN has a slow action potential, because Na_v_1.5 is not expressed in the SAN [2]. To test whether the age-dependent increase in pacemaker tissue with a slow action potential is the result of an increase in Na_v_1.5-negative tissue, cryosections were cut (in plane of short axis of heart) from the superior towards the inferior vena cava. For young and old animals, 10 to 15 sections at 200 μm intervals were immunolabelled for Na_v_1.5 and adjacent sections were immunolabelled for HCN4. Fig. 3 shows Na_v_1.5 labelling and Fig. 4 shows HCN4 labelling at the level of the superior vena cava in young and old animals. Na_v_1.5 was expressed in much of the tissue section (Figs. 3A and B) – it was expressed in the cell membrane as shown in the high-magnification image in Fig. 3C. However, in one region, Na_v_1.5 was not expressed (outlined with dotted white line in Figs. 3A and B). In contrast, HCN4 was only expressed in one region (outlined with dotted white line in Figs. 4A and B); in this region, it was expressed in the cell membrane as shown in Fig. 4C. Comparison of Figs. 3 and 4 shows that the tissue expressing Na_v_1.5 lacked expression of HCN4 and was, therefore, atrial muscle, whereas the tissue lacking expression of Na_v_1.5 expressed HCN4 and was, therefore, nodal. The Na_v_1.5-negative/HCN4-positive region in Figs. 3 and 4 was larger in the old animal. The Na_v_1.5-negative/HCN4-positive region also did not express the gap junction channel, Cx43 (data not shown).

**Fig. 5:**
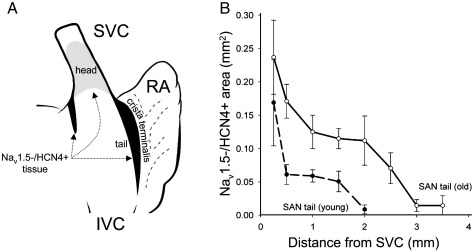
(A) Dorsal view of right atrium. SAN is shaded and forms inverted ‘U’. (B) Mean ( ± SEM; *n* = 5) area of Na_v_1.5-negative/HCN4-positive tissue at different levels of main SAN tail in young and old animals. Area plotted against distance of tissue section from superior vena cava (SVC). IVC, inferior vena cava; RA, right atrial free wall.

The shaded areas in Fig. 5A show schematically the distribution of Na_v_1.5-negative/HCN4-positive SAN tissue. The SAN is made up of a large ‘head’ located in the anterior wall of the superior vena cava and a tail extending down the side of the crista terminalis (and centred on SAN artery; Fig. 5A). There is a second smaller tail extending down the groove between the right and left atria (Fig. 5A). We have previously shown HCN4-positive tissue to be distributed in this manner in the young rat [15]. The sections shown in Figs. 3 and 4 are from the head. In old animals, the Na_v_1.5-negative/HCN4-positive region was larger in the main tail as well as head of the SAN: Fig. 5B shows the area of the Na_v_1.5-negative/HCN4-positive region in the main tail at different distances from the superior vena cava. Fig. 5B shows that the main tail was also longer in the old animals; in young and old animals, it was 45% and 57%, respectively, of the distance between the superior and inferior vena cava (Table 1).

The total volume of Na_v_1.5-negative/HCN4-positive tissue in the head and the two tails was estimated from data like those in Fig. 5B and was 97% greater in old animals (Table 1). The total volume of Na_v_1.5-negative/HCN4-positive tissue as a percentage of the volume of the heart (estimated from heart weight) did not change significantly with age (0.04 ± 0.007%, *n* = 5, and 0.05 ± 0.003%, *n* = 5, in young and old animals, respectively).

### 3.4 Why is the leading pacemaker site located in the tail of SAN?

Why is the leading pacemaker site always located in the main tail of Na_v_1.5-negative/HCN4-positive tissue next to the crista terminalis rather than the large head of Na_v_1.5-negative/HCN4-positive tissue in the ventral wall of the superior vena cava? The functional importance of the head and tail of the SAN was investigated in six right atrial preparations from young animals. In the intact preparations, the spontaneous cycle length was measured and the position of the leading pacemaker site was determined—the leading pacemaker site was located in the tail of the SAN next to the crista terminalis as observed previously. The superior vena cava was then separated from the right atrium in order to separate the SAN head from the SAN tail. In all six cases, the remaining right atrium (corresponding to SAN tail) continued to show spontaneous activity (spontaneous cycle length was shorter than in intact preparations – Data Supplement, Fig. S2) and the leading pacemaker site was shifted inferiorly (data not shown). In only two of the cases did the superior vena cava (corresponding to SAN head) show spontaneous activity – furthermore, the mean spontaneous cycle length of these two preparations was longer than that of the remaining right atrium (Data Supplement, Fig. S2). Isoproterenol, at 0.05 μM, decreased the spontaneous cycle length of both the two superior vena cava preparations that showed spontaneous activity as well as all the remaining right atrial preparations (Data Supplement, Fig. S2). Finally, when recording intracellular action potentials from the SAN head, we were only able to record pacemaker action potentials from one of four preparations (from old animals). It is concluded that the SAN tail and not the SAN head shows robust pacemaking and this is why the leading pacemaker site is always located in the SAN tail. Why this should be the case is not known.

### 3.5 Age-dependent enlargement of Na_v_1.5-negative/HCN4-positive tissue can be explained by hypertrophy of SAN cells

**Fig. 6:**
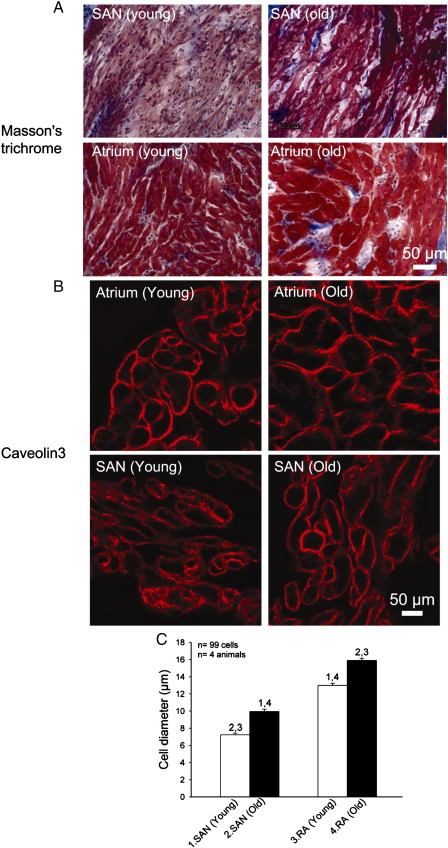
Change in cell diameter with age. (A) High-magnification images of SAN and atrial muscle (stained with Masson's trichrome) from young and old animals. (B) Immunolabelling of caveolin3 in SAN and atrial muscle in young and old animals. Caveolin3 is expressed in cell membrane in both SAN and atrial muscle. From images such as these, we measured cell diameter. (C) Mean (+ SEM; *n* = 4 animals; ∼ 25 cells/animal measured) cell diameter of SAN and atrial cells in young and old animals. Numbers denote statistically significant differences (P < 0.05) from appropriately numbered bars (one-way ANOVA).

Fig. 6A shows high-magnification images of Masson's trichrome stained sections of the SAN and atrial muscle (different tissue types were identified by characteristic pattern of expression of Na_v_1.5 and HCN4 in adjacent tissue sections) in a young and old animal – it shows that the SAN cells were smaller than the atrial cells and both cell types were larger in the old animal. To confirm this, sections were immunolabelled for caveolin3 to highlight the cell membrane in order to measure the cell diameter (Fig. 6B). In old animals, cell diameter was significantly increased by 38% and 23% in the SAN and atrial muscle, respectively (Fig. 6C). The total volume of the SAN is proportional to the SAN cell volume (if the number of SAN cells is constant) and cell volume is proportional to the square of the cell radius (if cell length remains unchanged). Calculation shows that the increase in SAN cell diameter of 38% is expected to increase the volume of the SAN by 90% and this is similar to that observed experimentally (97%).

### 3.6 Age-dependent remodelling of interstitium

**Fig. 7:**
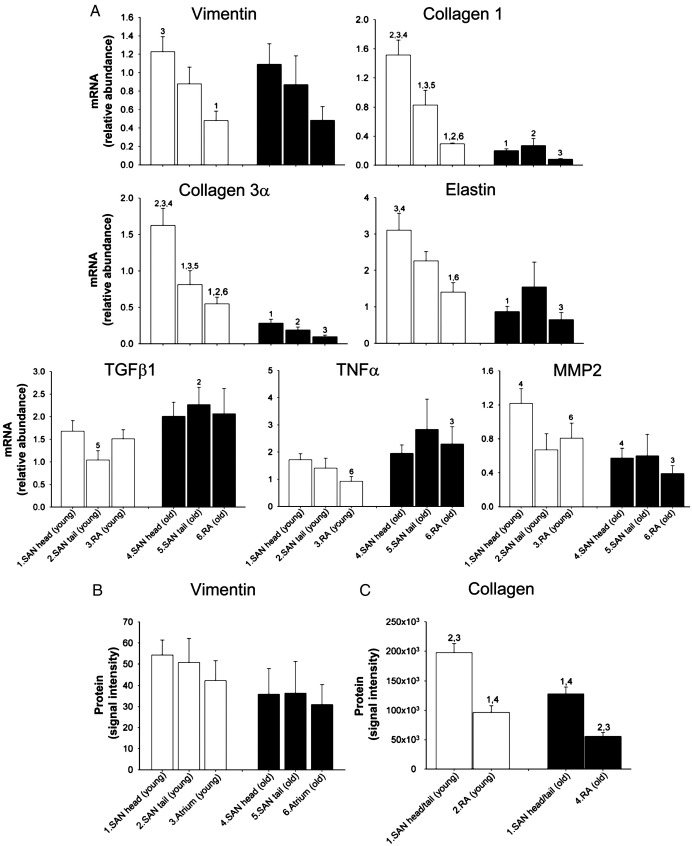
Age-dependent remodelling of interstitium. (A) Relative abundance of mRNAs for various components of interstitium in SAN and atrial muscle of young and old animals. Means + SEM (*n* = 8) shown. (B) Mean (+ SEM; *n* = 4) density of vimentin protein immunolabelling in SAN and atrial muscle of young and old animals. (C) Mean (+ SEM; *n* = 4) density of Picro Sirius red staining of collagen in SAN and atrial muscle of young and old animals. Numbers denote statistically significant differences (P < 0.05) from appropriately numbered bars (two-way ANOVA). RA, right atrial free wall.

**Fig. 8:**
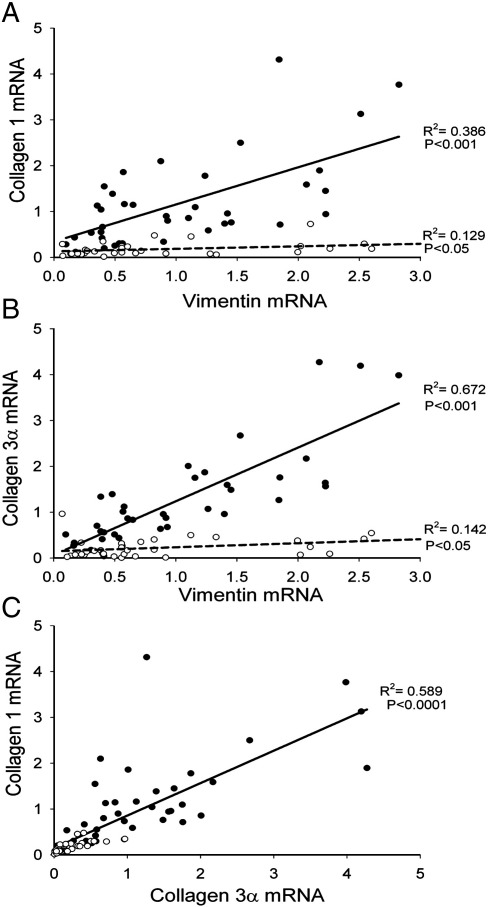
(A, B) Relationship between relative abundance of collagen 1 (A) or collagen 3 (B) mRNA and relative abundance of vimentin mRNA in young (filled symbols) and old (open symbols) animals. (C) Relationship between relative abundance of collagen 1 mRNA and relative abundance of collagen 3 mRNA. Data from eight young and old animals and three tissue types shown. Data fit with straight lines by linear regression (*R*^2^ and P values shown).

To test if there is an age-dependent remodelling of the interstitium of the SAN during ageing, the abundance of mRNAs for important interstitial components was measured using qPCR. The two main components of the interstitium are fibroblasts and the extracellular matrix (principally collagens) [9]. Vimentin is expressed by fibroblasts (as well as other cell types) [16]; as expected, vimentin mRNA was more abundant in the SAN than in the atrial muscle (Fig. 7A). This is consistent with the fibroblasts being more numerous in the SAN than in the atrial muscle. However, there was no age-dependent change in vimentin mRNA (Fig. 7A). Immunolabelling of vimentin (Data Supplement, Fig. S3) also revealed no age-dependent change in vimentin at the protein level (Fig. 7B). These data suggest that there was no age-dependent change in the number of fibroblasts in the SAN. Collagens are structural proteins made by the fibroblasts. Types 1 and 3 collagens constitute 80% of the total collagen present in the interstitium in the heart [17]. In young animals, like vimentin mRNA, mRNAs for types 1 and 3 collagens were more abundant in the SAN than in the atrial muscle (Fig. 7A). Because mRNAs for vimentin and types 1 and 3 collagens are produced by the fibroblasts, it is possible that they will vary together. In Figs. 8A and B, the filled symbols (and solid lines) show the relationship between the abundance of mRNA for types 1 and 3 collagens and the abundance of vimentin mRNA in young animals (each point corresponds to different animal or region – SAN head, SAN tail, or atrial muscle); there is a significant correlation as expected. However, unlike vimentin mRNA, there was a substantial and significant age-dependent decrease of mRNAs for types 1 and 3 collagens (Fig. 7A). In Figs. 8A and B, the open symbols (and dashed lines) show that there is still a significant correlation between the abundance of collagen and vimentin mRNAs in the old animals, but the relationship is depressed as compared to that in the young animals. As a result of these changes, in the old animals, the *ratio* of collagen type 1 mRNA to vimentin mRNA was depressed (Data Supplement, Fig. S4). The density of total collagen *protein* was determined by Picro Sirius red staining of sections from young and old animals (Data Supplement, Fig. S3). This showed that the density of collagen protein was higher in the SAN than in the atrial muscle as expected, and in both tissues, there was a significant age-dependent decrease in the density of collagen protein (Fig. 7C); this is consistent with the mRNA data (Fig. 7A). It has been reported that the ratio of collagen 1 (which is stiff) to collagen 3 (which is elastic) can change in disease [18]. For example, in diseased human ventricle, the proportion of collagen 1 increases, whereas the proportion of collagen 3 decreases, and this explains the increase in passive myocardial stiffness [19]. Fig. 8C shows the relationship between collagens 1 and 3 in the SAN and atrial muscle of the young and old animals; all the data lie along a single straight line and this indicates that *the ratio of collagen 1 to collagen 3 does not change with age* in the rat heart.

Another structural protein of the extracellular matrix is elastin. Once again, elastin mRNA was more abundant in the SAN than in the atrial muscle (Fig. 7A). As in the case of the collagen mRNAs, there was an age-dependent decrease in elastin mRNA (Fig. 7A). The ratio of collagen 1 mRNA to elastin mRNA was roughly constant and it did not vary significantly with region or age (Data Supplement, Fig. S4). Transforming growth factor β1 (TGFβ1) and tumour necrosis factor α (TNFα) are fibrotic agents: they promote the formation of extracellular matrix including collagens (regulated at transcriptional level) [20,21]. The abundance of TGFβ1 and TNFα mRNAs was similar in all tissues (Fig. 7A). There was a significant, but modest, age-dependent upregulation of TGFβ1 and TNFα mRNAs in one of the tissues at least (Fig. 7A). Metalloproteinases (MMPs) are a group of enzymes that catalyse the degradation of the extracellular matrix including collagens [22]. MMP2 is an important cardiac metalloproteinase [23]. The abundance of MMP2 mRNA was similar in all tissues and there was a significant age-dependent decrease in MMP2 mRNA in the SAN head and atrial muscle at least (Fig. 7A). In summary, these data provide a complex picture of age-dependent remodelling of the extracellular matrix: a modest increase in the abundance of fibrotic agents and a modest decrease in MMP2 (at mRNA level), both of which will promote fibrosis, and yet a decrease in abundance of mRNAs responsible for structural elements of the extracellular matrix.

In addition to the above, we observed no age-dependent change in a variety of other extracellular matrix components at the mRNA level: fibronectin α1 (adhesive protein), decorin (anti-fibrotic proteoglycan), connective tissue growth factor (CTGF; cysteine-rich protein induced by TGFβ1 and able to trigger many cellular processes underlying fibrosis), and integrins α1, α5, and β1 (membrane receptors, which mediate cell–extracellular matrix interactions [24]; data not shown). MMP1 (a and b types), MMP9 and MMP13 mRNAs were undetectable. Li et al. [25] also reported that MMP1 is undetectable in rat heart.

## 4 Discussion

This study has shown that the age-dependent decline in SAN function is associated with a structural remodelling of the SAN: an expansion of Na_v_1.5-negative/HCN4-positive tissue, a hypertrophy of the SAN cells and a remodelling of the extracellular matrix.

### 4.1 Age-dependent change in SAN function

The intrinsic heart rate was slower (i.e. intrinsic cycle length was longer) in the old animals (Data Supplement, Fig. S1). The same occurs in the human: although the normal heart rate (under control of autonomic tone) does not change with age, the intrinsic heart rate (heart rate in the absence of autonomic influence) declines from 107 beats/min at 25 years of age to 70 beats/min at 85 years of age [26]. The same decline has been observed in a variety of mammalian species [3].

### 4.2 Age-dependent enlargement of SAN

Intracellular action potential recording demonstrated an age-dependent increase in the extent of pacemaker tissue with a slow action potential in the superior-inferior axis (Table 1). This is qualitatively consistent with the study of Alings and Bouman [3], who showed that with age the surface area of pacemaker tissue with a slow action potential increases from 0.20 to 5.48 mm^2^ in the rabbit and from 0.04 to 2.08 mm^2^ in the cat. The upstroke of the SAN action potential is slow, because SAN tissue lacks *I*_Na_ and Na_v_1.5 and this study showed that the age-dependent increase in pacemaker tissue with a slow action potential is likely to be a consequence of a similar increase in the tissue lacking expression of Na_v_1.5 (Table 1). In the present study, in both young and old animals, the tissue that lacked expression of Na_v_1.5 expressed HCN4 (Figs. 3 and 4), but not Cx43 (data not shown)—Cx43 is a gap junction channel known to be present in the working myocardium, but absent from the SAN. It follows, therefore, that there was an age-dependent increase of Na_v_1.5-negative/HCN4-positive/Cx43-negative tissue. In the guinea pig, we have previously shown an age-dependent increase in SAN tissue lacking expression of Cx43 and Ca_v_1.2 (major L-type Ca^2+^ channel isoform in working myocardium) [27,28]. Interestingly, in the guinea pig, the increase of the SAN is greater than can be explained by any increase in heart size [27]. This study has shown that the enlargement of the SAN can be explained by a hypertrophy of the SAN cells (Fig. 6).

What are the functional correlates of the increase in the SAN? In the young animals, with a higher intrinsic heat rate, the leading pacemaker site was located near the superior vena cava, whereas in the old animals, with a lower intrinsic heart rate, the leading pacemaker site was located more inferiorly nearer the inferior vena cava (Table 1). This is consistent with the extension of pacemaker tissue towards the inferior vena cava in the old animals (Table 1). It is also consistent with the concept of the SAN containing a hierarchy of pacemakers: Boineau et al. [29] have suggested that within the SAN there is a hierarchy of pacemakers and, the more inferior the position in the SAN, the slower the heart rate. The increase in SAN tissue lacking expression of Na_v_1.5 possibly could help explain the increase in the SAN conduction time observed in the elderly and SAN exit block in sick sinus syndrome [30] if the SAN action potential has to propagate a greater distance from the leading pacemaker site in the SAN through tissue lacking Na_v_1.5 to reach the surrounding atrial muscle [2]. Blockade of Na_v_1.5 (e.g. Fig. 1), knockout of Na_v_1.5 and loss of function mutations in Na_v_1.5 also cause bradycardia [2,4]. However, the increase in SAN tissue lacking expression of Na_v_1.5 cannot explain the age-dependent decrease in the intrinsic heart rate, because blockade of Na_v_1.5 by 2 μM TTX had a greater effect on the heart rate in the old animals (Fig. 1). Blockade of HCN4 also had a greater effect on the heart rate in the old animal (Fig. 1). The greater sensitivity of SAN pacemaking in old animals to blockade of Na_v_1.5 and HCN4 possibly results from a shift in the relative contribution of different ionic mechanisms to pacemaking. If the decrease in the intrinsic heart rate during ageing is the result of another factor (e.g. decrease of L-type Ca^2+^ current [28] or ‘Ca^2+^ clock [31]), blockade of Na_v_1.5 and HCN4 may result in a larger effect in the old animal. This implies that there is a decrease in ‘pacemaking reserve’ in the old animal. If this is the case, a drug or naturally occurring mutation that results in a loss-of-function of an ion channel will cause a more serious bradycardia in the elderly.

### 4.3 Age-dependent remodelling of interstitium

Older literature claims that there is an age-dependent increase in the collagen protein content of the SAN and this is responsible for the decline in SAN function [8]. However, Alings et al. [8] criticised these studies, because of the non-specificity of the dyes used for collagen measurement. Alings et al. [8] used Picro Sirius red to measure collagen protein and they reported that in the human SAN, from adulthood, there is no age-dependent increase in collagen protein and there is also no age-dependent change in the cat SAN. Nevertheless, an age-dependent increase in the interstitium and a loss of nodal cells has recently been reported in the rat [9].

This is the first study to investigate the effects of ageing on the components of the interstitium. In the present study, in the SAN of old animals, there was no overt evidence of an increase of the interstitium and a loss of nodal cells in Masson's trichrome stained sections (Fig. 6A; cf [27]). The two main components of the interstitium are fibroblasts and collagen (made by fibroblasts) [9]. Consistent with the observations from the Masson's trichrome stained sections, there was no age-dependent change in vimentin mRNA and protein (fibroblast marker; Fig. 7) and, in fact, there was a decrease in collagen protein as measured by Picro Sirius red (Fig. 7B). The abundance of collagen protein depends on the balance of the rate of its synthesis from its mRNA and the rate of its degradation by MMPs. Although there was a modest age-dependent decrease in MMP2 mRNA (Fig. 7A; tending to result in increase in collagen protein), there was a large age-dependent decrease of mRNAs for types 1 and 3 collagen of ∼ 79% (Fig. 7A; tending to result in decrease in collagen protein) and presumably the latter effect dominated to explain the observed age-dependent decrease in collagen protein (Fig. 7B). The age-dependent decrease in collagen mRNAs occurred despite a modest age-dependent increase in TGFβ1 and TNFα mRNAs (Fig. 7A).

In contrast to the SAN, there appears to be a dramatic age-dependent increase in the interstitium and collagen protein content in the rat ventricles [32,33]. For example, from 3- to 24-month-old rats, Besse et al. [33] reported a 346% increase in collagen protein in the ventricles. Despite this possible difference between the SAN and ventricles, the underlying changes in the two tissues are likely to be the same. Consistent with the changes observed in the present study, from 3- to 24-month-old rats, Besse et al. [33] observed decreases of mRNAs for types 1 and 3 collagen of ∼ 50% in the ventricles. Consistent with this and the results from the present study, from 1- to 24-month-old rats, Mays et al. [34] observed a 10-fold decrease in the rate of collagen protein synthesis in the heart (whole); they observed similar changes in the lung and skin. Again consistent with the changes observed in the present study, from young to 24-month-old rats, Robert et al. [35] observed a 40% decrease in MMP2 activity and a corresponding decrease of MMP2 mRNA in the ventricles. In summary, there are age-dependent decreases of both collagen and MMP mRNAs. It is possible that, in the ventricles, the decrease in MMP mRNA is the dominating influence and, consequently, there is an age-dependent increase in collagen protein, whereas, in the SAN, the decreases of collagen and MMP mRNAs are more balanced and there can be no age-dependent change in collagen protein [8] or even a decrease as in the present study.

It is concluded that the age-dependent decline in SAN function is associated with a structural remodelling of the SAN: an increase in SAN tissue, a hypertrophy of the SAN cells and a remodelling of the extracellular matrix. The implications of the structural remodelling associated with ageing and/or obesity will require further investigation.

## Figures and Tables

**Table 1:** Characteristics relating to SAN in young and old animals.

	Young animals	Old animals	*t* test
Distance from superior to inferior vena cava	4.5 ± 0.3 mm (*n* = 7)	4.7 ± 0.3 mm (*n* = 6)	Not significant
Distance of leading pacemaker site from superior vena cava	1.0 ± 0.4 mm (*n* = 7)	2.5 ± 0.4 mm (*n* = 6)	P < 0.05
Extent of pacemaking tissue along SAN artery	1.7 ± 0.2 mm (*n* = 6)	3.1 ± 0.3 mm (*n* = 7)	P < 0.01
Length of Na_v_1.5-negative/HCN4-positive main tail of SAN	2 ± 0.2 mm (*n* = 5)	2.7 ± 0.3 mm (*n* = 5)	P < 0.05
Total volume of Na_v_1.5-negative/HCN4-positive tissue in SAN head and tails	0.48 ± 0.06 mm^3^ (*n* = 5)	0.94 ± 0.07 mm^3^ (*n* = 5)	P < 001

Means  ±  SEM (*n*) are shown.

## References

[1] Cheitlin M.D. (2003). Cardiovascular physiology—changes with aging. Am. J. Geriatr. Cardiol..

[2] Dobrzynski H., Boyett M.R., Anderson R.H. (2007). New insights into pacemaker activity: promoting understanding of sick sinus syndrome. Circulation.

[3] Alings A.M.W., Bouman L.N. (1993). Electrophysiology of the ageing rabbit and cat sinoatrial node—a comparative study. Eur. Heartf J..

[4] Zhang H., Zhao Y., Lei M., Dobrzynski H., Liu J.H., Holden A.V. (2007). Computational evaluation of the roles of Na^+^ current, *I*_Na_, and cell death in cardiac pacemaking and driving. Am. J. Physiol..

[5] Burgess M.L., McCrea J.C., Hedrick H.L. (2001). Age-associated changes in cardiac matrix and integrins. Mech. Ageing Dev..

[6] Boyett M.R., Honjo H., Kodama I. (2000). The sinoatrial node, a heterogeneous pacemaker structure. Cardiovasc. Res..

[7] Masson-Pévet M.A., Bleeker W.K., Besselsen E., Treytel B.W., Jongsma H.J., Bouman L.N. (1984). Pacemaker cell types in the rabbit sinus node: a correlative ultrastructural and electrophysiological study. J. Mol. Cell. Cardiol..

[8] Alings A.M., Abbas R.F., Bouman L.N. (1995). Age-related changes in structure and relative collagen content of the human and feline sinoatrial node. A comparative study. Eur. Heart. J..

[9] de Melo S.R., de Souza R.R., Mandarim-de-Lacerda C.A. (2002). Stereologic study of the sinoatrial node of rats—age related changes. Biogerontology.

[10] J.O. Tellez, M. Maczewski, P.V. Sutyagin, U. Mackiewicz, J. Yanni, A. Atkinson, et al. Ageing-dependent remodelling of ion channel and Ca^2+^ clock genes underlying sinoatrial node pacemaking. (submitted for publication) 2009. 10.1113/expphysiol.2011.057752 21724736

[11] Lei M., Jones S.A., Liu J., Lancaster M.K., Fung S.S., Dobrzynski H. (2004). Requirement of neuronal- and cardiac-type sodium channels for murine sinoatrial node pacemaking. J. Physiol..

[12] Nikmaram M.R., Boyett M.R., Kodama I., Suzuki R., Honjo H. (1997). Variation in the effects of Cs^+^, UL-FS 49 and ZD7288 within the sinoatrial node. Am. J. Physiol..

[13] Bois P., Bescond J., Renaudon B., Lenfant J. (1996). Mode of action of bradycardic agent, S 16257, on ionic currents of rabbit sinoatrial node cells. Br. J. Pharmacol..

[14] Sutyagin P.V., Kalinina E.E., Pylaev A.S. (2005). Morphofunctional organization of sinoatrial node in rat heart. Bull. Exp. Biol. Med..

[15] Yamamoto M., Dobrzynski H., Tellez J., Niwa R., Billeter R., Honjo H. (2006). Extended atrial conduction system characterized by the expression of the HCN4 channel and connexin45. Cardiovasc. Res..

[16] Laurila P., Virtanen I., Lehto V.P., Vartio T., Stenman S. (1982). Expression and distribution of vimentin and keratin filaments in heterokaryons of human fibroblasts and amnion epithelial cells. J. Cell. Biol..

[17] Weber K.T. (1989). Cardiac interstitium in health and disease: the fibrillar collagen network. J. Am. Coll. Cardiol..

[18] Weber K.T., Brilla C.G., Janicki J.S. (1993). Myocardial fibrosis: functional significance and regulatory factors. Cardiovasc. Res..

[19] Bishop J.E., Greenbaum R., Gibson D.G., Yacoub M., Laurent G.J. (1990). Enhanced deposition of predominantly type I collagen in myocardial disease. J. Mol. Cell. Cardiol..

[20] Abraham D.J., Shiwen X., Black C.M., Sa S., Xu Y., Leask A. (2000). Tumor necrosis factor alpha suppresses the induction of connective tissue growth factor by transforming growth factor-beta in normal and scleroderma fibroblasts. J. Biol. Chem..

[21] Susic D., Frohlich E.D. (2008). The aging hypertensive heart: a brief update. Nat. Clin. Pract. Cardiovasc. Med..

[22] Malemud C.J. (2006). Matrix metalloproteinases (MMPs) in health and disease: an overview. Front. Biosci..

[23] Robert V., Besse S., Sabri A., Silvestre J.S., Assayag P., Nguyen V.T. (1997). Differential regulation of matrix metalloproteinases associated with aging and hypertension in the rat heart. Lab. Invest.fs.

[24] Takada Y., Ye X., Simon S. (2007). The integrins. Genome. Biol..

[25] Li H., Simon H., Bocan T.M., Peterson J.T. (2000). MMP/TIMP expression in spontaneously hypertensive heart failure rats: the effect of ACE- and MMP-inhibition. Cardiovasc. Res..

[26] Jones S.A. (2006). Ageing to arrhythmias: conundrums of connections in the ageing heart. J. Pharm. Pharmacol..

[27] Jones S.A., Lancaster M.K., Boyett M.R. (2004). Ageing-related changes of connexins and conduction within the sinoatrial node. J. Physiol..

[28] Jones S.A., Boyett M.R., Lancaster M.K. (2007). Declining into failure: the age-dependent loss of the L-type calcium channel within the sinoatrial node. Circulation.

[29] Boineau J.P., Schuessler R.B., Roeske W.R., Autry L.J., Miller C.B., Wylds A.C. (1983). Quantitative relation between sites of atrial muscle origin and cycle length. Am. J. Physiol..

[30] Benditt D.G., Sakaguchi S., Goldstein M.A., Lurie K.G., Gornick C.C., Adler S.W., Zipes DP, Jalife J (1995). Sinus node dysfunction: pathophysiology, clinical features, evaluation, and treatment. Cardiac electrophysiology: from cell to bedside.

[31] Bogdanov K.Y., Vinogradova T.M., Lakatta E.G. (2001). Sinoatrial nodal cell ryanodine receptor and Na^+^–Ca^2+^ exchanger: molecular partners in pacemaker regulation. Circ. Res..

[32] Mays P.K., Bishop J.E., Laurent G.J. (1988). Age-related changes in the proportion of types I and III collagen. Mech. Ageing Dev..

[33] Besse S., Robert V., Assayag P., Delcayre C., Swynghedauw B. (1994). Nonsynchronous changes in myocardial collagen mRNA and protein during aging: effect of DOCA-salt hypertension. Am. J. Physiol..

[34] Mays P.K., McAnulty R.J., Campa J.S., Laurent G.J. (1991). Age-related changes in collagen synthesis and degradation in rat tissues. Importance of degradation of newly synthesized collagen in regulating collagen production. Biochem. J..

[35] Robert V., Besse S., Sabri A., Silvestre J.S., Assayag P., Nguyen V.T. (1997). Differential regulation of matrix metalloproteinases associated with aging and hypertension in the rat heart. Lab. Invest..

