# Supplementary material for: Structural remodelling of the sinoatrial node in obese old rats
Source: J Mol Cell Cardiol. 2010 Apr;48(4):653–62. doi: 10.1016/j.yjmcc.2009.08.023 (PMC2845824; doi:10.1016/j.yjmcc.2009.08.023)
Supplement: Supplementary data [file mmc1.doc]

DATA SUPPLEMENT

**Structural remodelling of the sinoatrial node in obese old rats**

J. Yanni, J. Tellez, P.V. Sutyagin, M.R. Boyett, H. Dobrzynski

**Methods**

**Tissue preparation**

Male Wistar-Hanover rats aged three and 24 months were used (electrophysiology, n=26; histology and immunohistochemistry, n=10; quantitative PCR, n=16). All animal procedures were carried out in accordance with the United Kingdom Animal (Scientific Procedures) Act 1986. Rats were weighed and then killed by intraperitoneal injection of phenobarbital (60 mg/kg). The heart was rapidly removed from the chest and placed in cold (4°C) Krebs-Ringer solution containing (in mM): NaCl, 100; KCl, 4; MgSO_4_, 1.2; KH_2_PO_4_, 1.2; CaCl_2_, 1.8; NaHCO_3_, 25; glucose, 10. The solution was bubbled with 95 % O_2_ and 5 % CO_2_ to give a pH of 7.4. Lung tissue and excess connective tissue were removed and then the hearts were weighed. For electrophysiology, the right atrium was isolated and it included the superior vena cava. Preparations were placed in a chamber and perfused with warm (37°C) Krebs-Ringer solution at a rate of 5 ml/min for electrophysiological recordings. For Masson’s trichrome and Picro-Sirius red staining and immunohistochemistry, the whole heart was embedded in OCT compound (Leica, UK) and frozen. Frozen serial sections (20 µm thickness) were cut in the short axis plane and sections were collected at 200 µm intervals. Sections were mounted on Superfrost glass slides (VWR, UK) and stored at -80°C until use. For RNA isolation, tissue samples (1 x 1 mm) were taken from the head and tail of the sinoatrial node (SAN) as well as the right atrial free wall. Samples from the SAN tail were taken approximately at the level of the leading pacemaker site in the SAN (at the level of the main branch from the crista terminalis). The samples were immersed in OCT and frozen in liquid N_2_.

**Electrophysiology**

Extracellular potentials were recorded by bipolar electrodes (consisting of two stainless steel wires, 100 µM in diameter, one electrode 1 mm shorter than the other, insulated to their tips and taped together) as described by Yamamoto et al. [1]. At the start of an experiment, an accurate drawing of a SAN/atrial muscle preparation was made with the help of a fine probe held by a calibrated micromanipulator with an accuracy of 0.1 mm. For mapping of electrical activity of the preparation, two pairs of modified bipolar electrodes were used. One pair of bipolar electrodes was used to record the extracellular potential from the atrial muscle and was used as a reference electrode. The other pair of bipolar electrodes was used to record the extracellular potential throughout the preparation at 0.5 mm intervals; the extracellular potential was recorded at 80 to 100 sites. The time of activation at the recording site was marked by a sharp deflection of the extracellular potential. The time interval between the time of activation at the reference electrode and the time of activation at the roving electrode was calculated. The point that showed the longest interval (earliest activation) was considered as the leading pacemaker site. The time of activation at other points was calculated with respect to the time of initiation of the action potential at the leading pacemaker site.

Intracellular action potentials were recorded using conventional microelectrodes filled with 3M KCl (the resistance of microelectrodes was ~30 MΩ). Electrical signals were digitised at 5 kHz by a Digitdata 1322A A/D converter (Axon Instruments Inc., Union city, USA) and stored on a computer for later analysis. Tetrodotoxin (TTX) was added to the Krebs-Ringer solution to block *I*_Na_ or CsCl or ivabradine was added to block *I*_f_.

**Masson’s trichrome**

Sections were stained with Masson’s trichrome as previously described to show the morphology [2]. With this technique, connective tissue was stained blue, cardiac myocytes were stained pink and nuclei were stained dark blue to black. Images of tissue sections were collected using a Zeiss light microscope and AxioVision software was used to collect and stitch the images.

**Picro Sirius red staining**

SAN sections were fixed in 10 % neutral buffered formalin for 30 min at room temperature. Sections were then washed in water three times (10 min each wash). Sections were placed into Picro-Sirius red solution for 1 h at room temperature. After that, sections were washed in acetic acid water twice (10 min each wash). The sections were dehydrated by placing them in different concentrations of alcohol as follows: 1 min in 70 % alcohol, 1 min in 90 % alcohol and 2 min in absolute alcohol. After dehydration, the sections were placed in clear solvent (xylene) twice (5 min each). Lastly, the sections were mounted in DPX mounting medium (Fluka). Images of tissue sections were collected using a polariz`ing microscope (collagen fibres appear red).

**Antibodies**

Primary antibodies (IgGs) were used for immunolabelling of sections. Mouse anti-Cx43 IgG raised against residues 252-270 of rat Cx43 (1:1000 dilution; Chemicon), rabbit anti-Na_v_1.5 IgG raised against residues 493-511 of rat Na_v_1.5 (1:50; dilution, Alomone labs), rabbit anti-HCN4 IgG raised against residues 119-155 of human HCN4 (1:50 dilution; Alomone labs), mouse anti-caveolin3 IgG raised against residues 3-24 of rat caveolin3 (1:1000; BD transduction), and guinea-pig anti-vimentin IgG raised against cow vimentin (1:500; Progen Biotechnik) were used. To detect the primary IgGs, secondary IgGs conjugated to fluorescence markers were used: donkey anti-mouse IgG conjugated to Cy3 (Chemicon) and donkey anti-rabbit IgG conjugated to FITC (Chemicon). No labelling above background was detected when either primary or secondary antibodies were used alone.

**Immunohistochemistry**

Immunohistochemistry was carried out using established methods as described previously [3]. Sections were fixed in 10 % buffered formalin for 30 min at room temperature. Sections were then washed with 0.01 M phosphate buffer saline (PBS) three times (10 min each wash). After that, the sections were treated by 0.1 % Triton-X100 for 30 min at room temperature. After treatment with Triton-X100, sections were washed with PBS three times (10 min each wash). Sections were then blocked with 1 % bovine serum albumin (BSA) in PBS for 1 h at room temperature. Sections were incubated with primary antibodies (diluted in 1 % BSA) at 4°C overnight. After incubation, sections were washed with PBS three times (10 min each wash). Sections were incubated with secondary antibodies for 1-2 h at room temperature. After incubation with secondary antibodies, sections were washed with PBS three times (10 min each wash). Finally, sections were mounted with Vectashield mounting medium (Vector Labs) and covered with coverslips. The coverslip edges were sealed with nail varnish.

**Measurement of immunofluorescence and Picro Sirius red signal intensity**

Data were collected for groups of four animals. Sections used to measure signal intensity were immunolabelled for vimentin or stained with Picro Sirius red. Sections to be compared were immunolabelled/stained in the same experiment under the same experimental conditions. For each labelled protein, high magnification images (×40 for immunolabelling and ×20 for Picro Sirius red staining) were collected using the same microscope parameters. 10-15 images per region per animal were collected. Velocity software (Improvision, UK) [4,5] was used to measure the signal intensity (in arbitrary units) for each protein. Signal intensity was only measured in user defined regions.

**RNA isolation and generation of cDNA**

The tissue samples were cut into 20 μm sections on a cryostat for RNA isolation. Total RNA was isolated from the frozen 20 μm sections of SAN and atrial muscle with a Qiagen muscle RNA extraction procedure (as previously described [6]). The isolated RNA (and resulting cDNA) samples from different rats were kept separate, i.e. the samples were not pooled. The RNA concentration was then measured with a Nanodrop ND-1000 spectrophotometer (Nanodrop Techologies, USA). 125 ng of total RNA from each sample was reverse transcribed with Superscript III reverse transcriptase (Invitrogen) in a 20 μl reaction according to the manufacturer’s instructions, using random hexamer priming. Aliquots of the resulting cDNA were diluted 10-fold in water for direct use in quantitative PCR (qPCR).

**Quantitative PCR**

qPCR was performed using an ABI Prism 7900 HT Sequence Detection System (Applied Biosystems, Foster, USA). The reaction mixture was comprised of 1 μl of cDNA, 900 nM forward primer, 900 nM reverse primer or 1x Qiagen assay (see Tables S1 and S2), 1x Syber Green Master Mix (Applied Biosystems) and DNAse-free water and the final volume was 10 μl. All samples were run in triplicate. The reaction conditions were: denaturation step of 95°C for 10 min, 40 cycles of amplification and quantification steps of 95°C for 30 s, 60°C for 30 s and 72°C for 1 min. The melt curve conditions were: 95°C for 15 s, 60°C for 15 s and 95°C for 15 s. By running a melting curve after the PCR reaction, the desired amplicon can be differentiated from other non-specific products. The size of qPCR products and specificity of primers were tested by running samples on 2 % agarose gels containing ethidium bromide and visualised by ultraviolet light. Gene expression was analysed by a double standardisation method (modified ΔΔCt method) described by Tellez et al. [6].

**Statistical analysis**

Data are presented as means±SEM and statistical differences assessed by Student’s *t* test, one way-ANOVA or two way ANOVA as appropriate. Differences were considered significant if P<0.05. n corresponds to the number of animals.

References

[1] Yamamoto M, Dobrzynski H, Tellez J, Niwa R, Billeter R, Honjo H, et al. Extended atrial conduction system characterised by the expression of the HCN4 channel and connexin45. Cardiovasc Res 2006;271-81.

[2] Dobrzynski H, Li J, Tellez J, Greener ID, Nikolski VP, Wright SE, et al. Computer three-dimensional reconstruction of the sinoatrial node. Circulation 2005;846-54.

[3] Dobrzynski H, Marples DD, Musa H, Yamanushi TT, Henderson Z, Takagishi Y, et al. Distribution of the muscarinic K^+^ channel proteins Kir3.1 and Kir3.4 in the ventricle, atrium, and sinoatrial node of heart. J Histochem Cytochem 2001;1221-34.

[4] Snelson CD, Santhakumar K, Halpern ME, Gamse JT. Tbx2b is required for the development of the parapineal organ. Development 2008;1693-702.

[5] Foster K, Sheridan J, Veiga-Fernandes H, Roderick K, Pachnis V, Adams R, et al. Contribution of neural crest-derived cells in the embryonic and adult thymus. J Immunol 2008;3183-9.

[6] Tellez JO, Dobrzynski H, Greener ID, Graham GM, Laing E, Honjo H, et al. Differential expression of ion channel transcripts in atrial muscle and sinoatrial node in rabbit. Circ Res 2006;1384-93.

**Table S1. Summary of commercially available primers used for quantitative PCR.**

| **Target transcript** | **Species** | **Qiagen catalogue**  **number** |
| --- | --- | --- |
| CTGF | Rat | QT00182021 |
| Decoin | Rat | QT01626842 |
| Elastin | Rat | QT01575924 |
| Fibronectin 1 | Rat | QT00179333 |
| Integrin alpha 1 | Rat | QT00193172 |
| Integrin alpha 5 | Rat | QT00431053 |
| Integrin beta 1 (fibronectin receptor beta) | Rat | QT00187656 |
| Matrix metallopeptidase 1a | Rat | QT01792350 |
| Matrix metallopeptidase 1b | Rat | QT01826748 |
| Matrix metallopeptidase 2 | Rat | QT00996254 |
| Matrix metallopeptidase 9 | Rat | QT00178290 |
| Matrix metallopeptidase 13 | Rat | QT01629593 |
| Procollagen, type III, alpha 1 | Rat | QT01083537 |
| Transforming growth factor, β1 receptor | Rat | QT00190953 |
| Vimentin | Rat | QT00178724 |

**Table S2. Summary of custom-designed primers used for quantitative PCR.**

| **Target transcript** | **Primer sequence 5′-3′** |
| --- | --- |
| 28S | F: GTTGTTGCCATGGTAATCCTGCTCAGTACG  R: TCTGACTTAGAGGCGTTCAGTCATAATCCC |
| Collagen type 1 | F: GCGAAGGCAACAGTGGATTC  R: GAGTCACACCGGAACTTGGG |
| Matrix metallopeptidase 9 | F: ACCCTGCGTATTTCCATTCATCT  R: AAGGTCGCTCGGATGGTTATC |
| Tumour necrosis factor alpha | F: GACAAGGCTGCCCCGACTA  R: AGGGCTCTTGATGGCAGAGA |


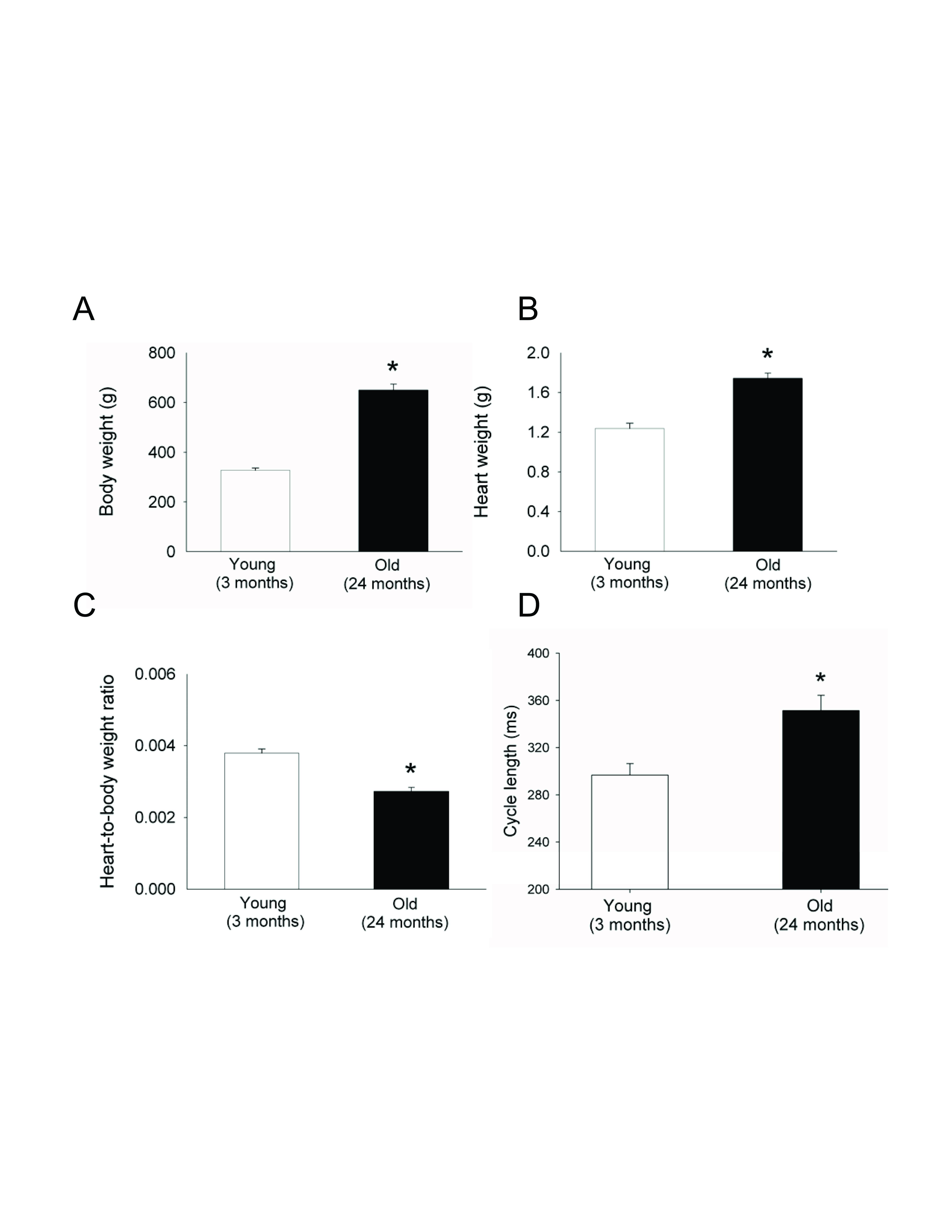


**Fig. S1. Comparison of young and old animals.** A-D, mean (+SEM) body weight (A; n=11), heart weight (B; n=11), heart-to-body weight ratio (C; n=11) and spontaneous cycle length (D; measured in right atrial preparations; n=7/6) of two age groups. *significantly different (P<0.001) from young animals (t test).


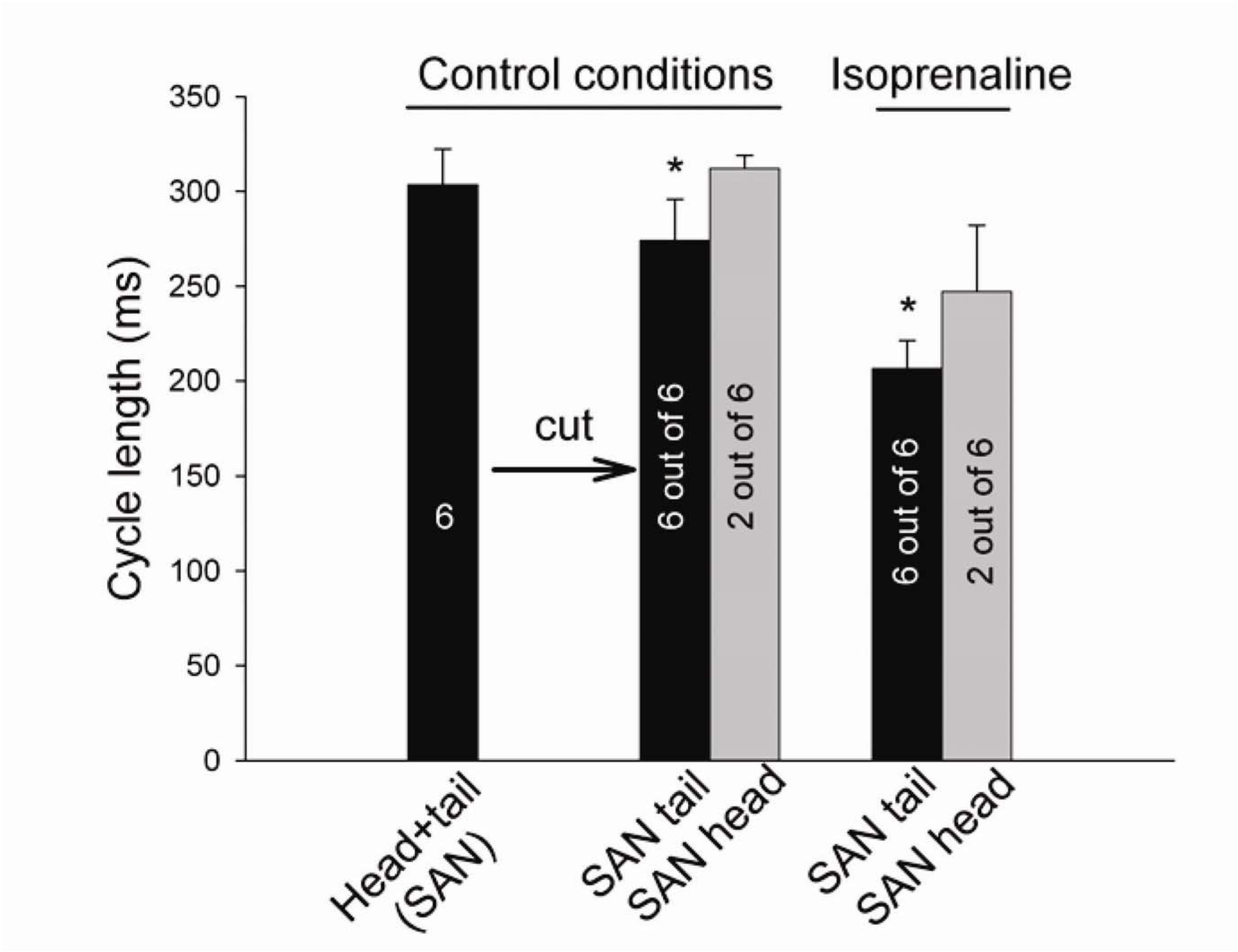


**Fig. S2. Effect of separating head from tail of SAN.** Left hand bar, mean (+SEM) spontaneous cycle length of six normal right atrial preparations. Middle pair of bars, mean (+SEM) spontaneous cycle length of tail (black) and head (grey) of SAN after separating two. Whereas n=6 for tail of SAN, it is only two for head. Right hand pair of bars, mean (+SEM) spontaneous cycle length of tail (black) and head (grey) of SAN after application of 0.05 μM isoprenaline. *significantly different (P<0.05) from left hand bar (paired t test).


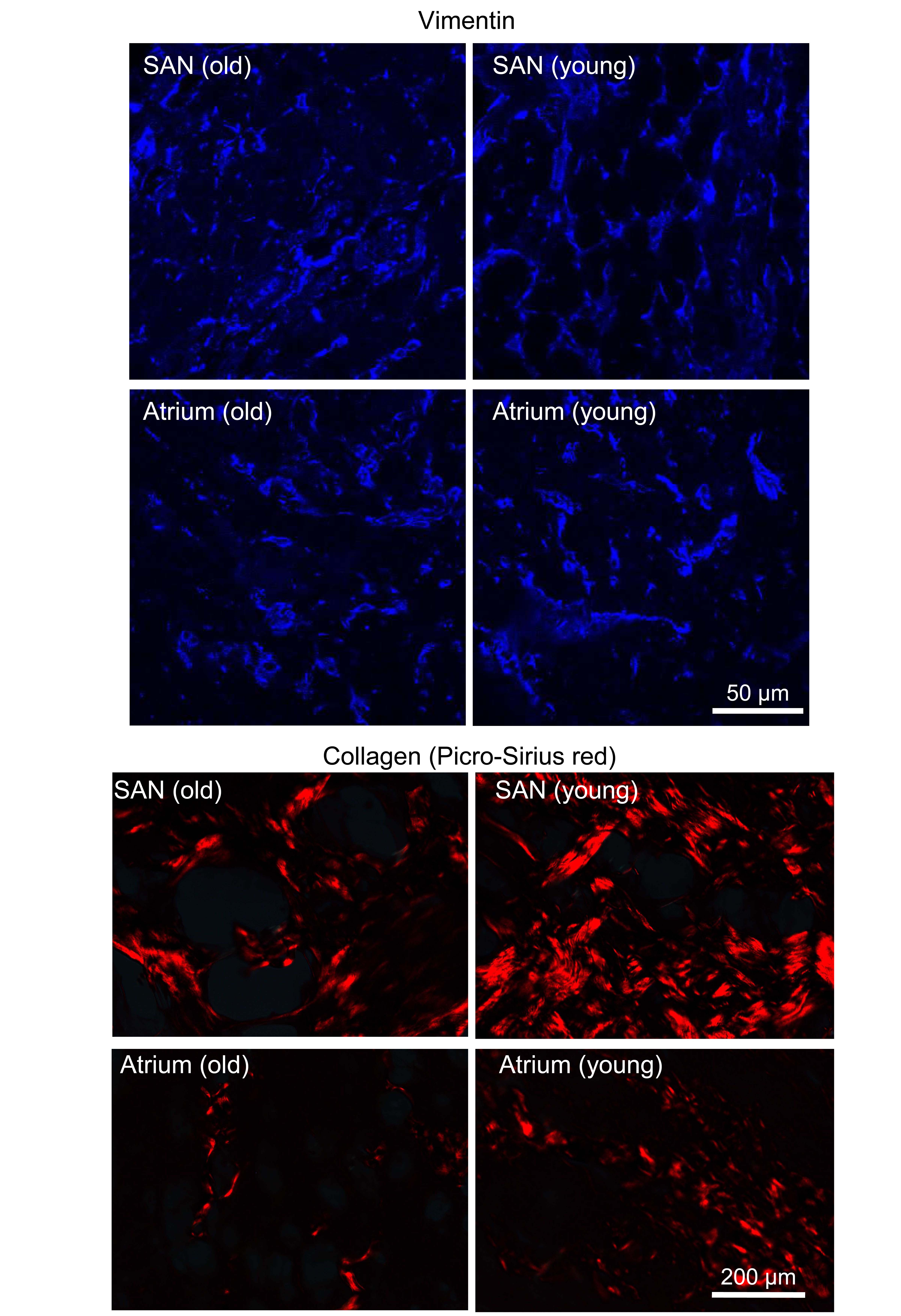


**Fig. S3. Comparison of vimentin (protein) and collagen (protein) labelling in young and old animals.** Top, immunolabelling of vimentin in SAN and atrial muscle of young and old animals. Bottom, Picro Sirius red staining of collagen in SAN and atrial muscle of young and old animals.


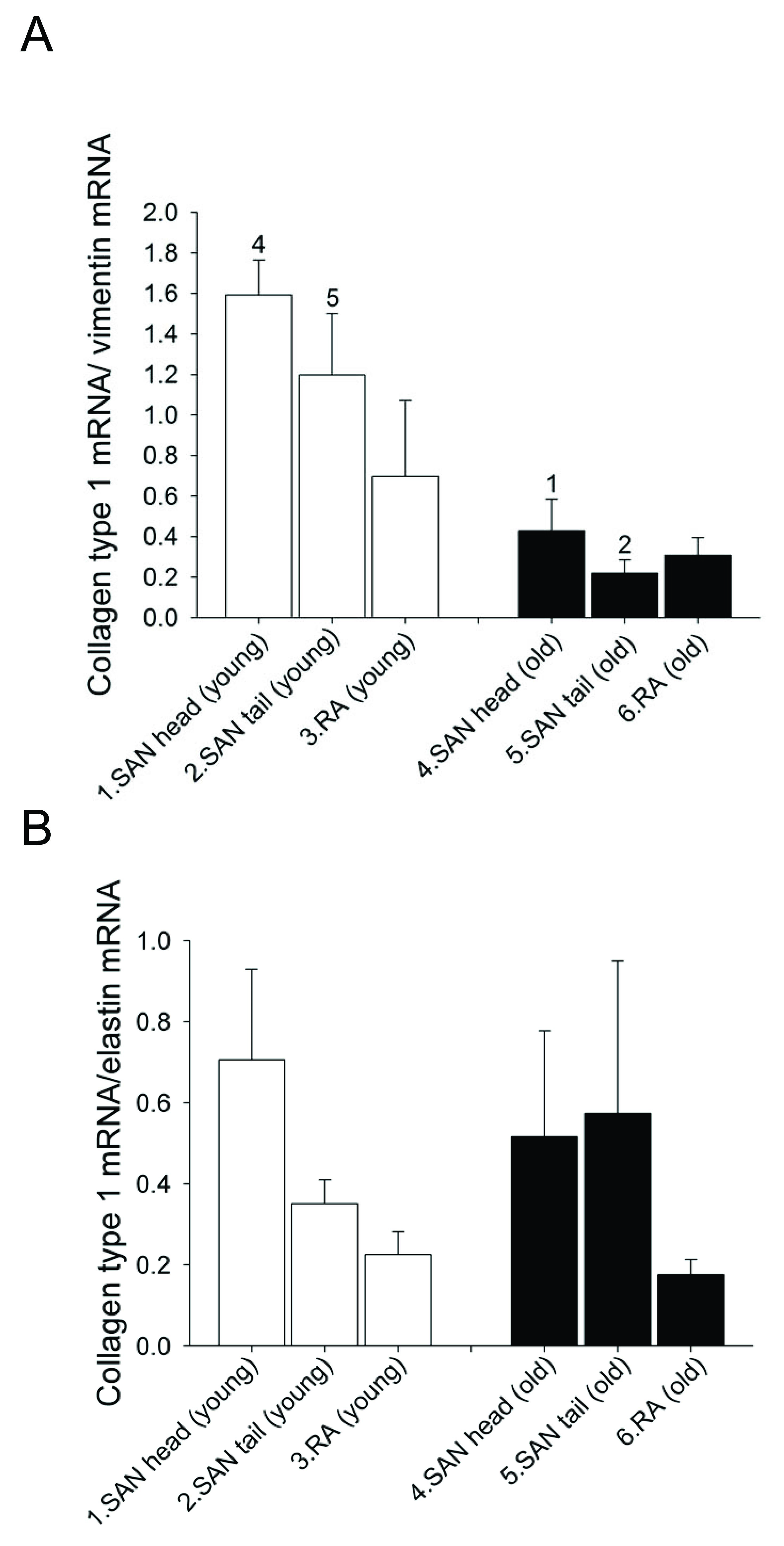


**Fig. S4.** A, ratio of collagen type 1 mRNA to elastin mRNA in young and old animals. B, ratio of collagen type 1 mRNA to vimentin mRNA in young and old animals. Means ± SEM shown (n=8). Numbers denote statistically significant differences (P<0.05) from the appropriately numbered bars (one-way ANOVA).
